# Opportunities for Brucellosis Control in Mexico: Views Based on the Sustainable Livelihoods Perspective

**DOI:** 10.3389/fvets.2019.00216

**Published:** 2019-07-02

**Authors:** David Oseguera Montiel, Klaas Frankena, Henk Udo, Akke van der Zijpp

**Affiliations:** ^1^Facultad de Medicina Veterrinaria y Zootecnia, Universidad Autónoma de Yucatán, Mérida, Mexico; ^2^Adaptation Physiology Group, Wageningen University, Wageningen, Netherlands; ^3^Animal Production Systems Group, Wageningen University, Wageningen, Netherlands

**Keywords:** Bajío, disease control, livelihoods, “One health”, smallholders, zoonosis, goats, participatory methods

## Abstract

Caprine brucellosis is a main constraint for small-scale goat husbandry systems in the Global South, as it negatively affects production parameters and can be transmitted to humans. The aim of this manuscript is to point out opportunities for brucellosis control in a resource poor area. The present paper draws from previous research in two Mexican states, Jalisco and Michoacán, both within the Bajío region. Main opportunities for brucellosis control are discussed within the “Sustainable Livelihoods Perspective.” Goat farming gives farmers a number of livelihoods benefits: food, cash, prestige, and a job. Goat farming is also a reason for some farmers to stay in their villages rather than to migrate to the US. This livelihood strategy, however, is threatened by brucellosis, which is endemic in the goat population of the region. Brucellosis control, however, offers an opportunity for small-scale goat farmers to enhance health and welfare. The socio-economic context is very important in planning a successful brucellosis control campaign. Control strategies should be planned considering the local goat farming husbandry and the views of the farmers.

## Introduction

Brucellosis is a common zoonotic disease worldwide (1). Despite the wealth of knowledge, including tools like vaccines and serological tests, brucellosis remains endemic in Mexico as in many other countries of the global South (2). Goats are the natural host of *Brucella melitensis*, which is considered to be the most virulent *Brucella* specie in humans. Caprine brucellosis is a great constraint for smallholder goat farming development and a threat for public health and for farmers' livelihoods (3). Although brucellosis is unlikely to be a fatal disease in humans, who are accidental hosts, it is a severe health condition, very debilitating, and disabling (4).

It causes high fever and may lead to complications, such as arthritis and spondylitis, abortion in pregnant women and orchitis in men (5, 6). Brucellosis infections are often not timely recognized as fever, an early symptom, is a clinical sign of many other infectious diseases. In developed countries, it can happen that physicians are not alert to suspect brucellosis in patients as brucellosis is not present in domestic livestock from many northern countries (7–9).

Disciplinary research with regard to brucellosis has been key for the control of brucellosis. Vaccines and serological testing are important technologies that have been used with great success in developed countries (10, 11). In the Global South, however, these technologies are not widely adopted by farmers and therefore brucellosis control is still ineffective. Smallholder farmers in the global South are embedded in complex context which is characterized first by the various purposes livestock husbandry has. First, keeping animals is a source of income, food, security, manure, prestige, and security among others. In addition livestock husbandry is often one of many other activities or strategies that farmers have. Second, strong governmental institutions are often lacking and third are prone to shocks, trends, and seasonality; which can be droughts, inflation, insecurity, and violence to mention some. Thus, strategies to improve livestock husbandry need to be in harmony with farmers' goals aiming to help them to better cope with a tough environment. The sustainable livelihoods framework can be an approach to capture the complexity of farmers livelihoods (12).

The aim of this paper is to explore feasible opportunities for brucellosis control in Mexico through the lens of the sustainable livelihoods perspectives. We argue that current control strategies for brucellosis in goats could have more impact if they are drawn from a liveslihoods perspective. This paper is organized as follows: as a background we first present a brief explanation of how brucellosis control is organized in Mexico and briefly show the current status of brucellosis in livestock control in Mexico and the incidence of human brucellosis in recent years. We then characterize the goat farming system and how goat farmers make a living. Finally an explanation of the linkages between goat husbandry and brucellosis endemicity is presented to show opportunities for brucellosis control based on farmers livelihoods.

## Brucellosis in Mexico: Policies and Progress to Control It

Brucellosis control in Mexico is based on a set of rules issued in 1995 (13). The “Norma Oficial Mexicana NOM−041-ZOO-1995” enlists the steps to control brucellosis. Three phases of brucellosis control are considered: (1) control, (2) eradication, and (3) free. Actions vary depending on the progress in a region as follows:

### Control

Applied to a region with high seroprevalence (>3%). The core strategy is to vaccinate all ruminants.

### Eradication

For a region with a low seroprevalence (<3%) the strategy focuses on test-and-cull of seropositive animals.

### Free

A region and also individual farms can obtain a free status recognition if the herds of cattle and flocks of small ruminants have been seronegative in 3 consecutive years. The core strategy then is continuous surveillance.

Only three municipalities of the west northern state Sonora are free of brucellosis. Sixteen municipalities have reached the eradication phase. The latter belong to 13 states scattered in central Mexico, in the south-east, western coast, and one state in the north coast. All other municipalities are in control phase (14).

The campaign for eradication and control for brucellosis is decentralized; each state has a committee for animal production, health, and welfare. Committees are funded by public resources. For the control of brucellosis, committees receive resources from the federal government to pay for staff salaries, vaccines against brucellosis, and biological reagents; i.e., Rose Bengal test to run serological analysis. The way each committee administrates its resources varies largely. Some committees hire their own field staff for vaccination and surveillance while other committees provide inputs for freelance veterinarians. Freelance veterinarians will offer their services to farmers and charge for their services to farmers.

## Livelihoods and Goat Husbandry

The sustainable livelihoods perspective is utilized to analyze how farmers make use of their capabilities, assets, and strategies to make a living and to cope with shocks, trends, and seasonality (12).

The five key capitals often considered in livelihoods perspectives are: (1) Natural capital; such as farmers' crop and grazing land. (2) Social capital; farmers' connections and working together patterns. (3) Financial capital; income, credits, and cash. (4) Human capital, people's health, age, gender, knowledge, and skills. (5) Physical capital, infrastructure, and the means to generate financial capitals such as goat flocks (15).

Within the livelihoods perspectives these five key capitals help people to withstand a vulnerability context where shocks, trends, and seasonality occur. A shock can be a disease outbreak, trends are for example, declining rural population and seasonality can refer to farming cycles; milking, lambing, cropping, harvesting, and others like, weather related cycles. Capitals are also the means by which people implement livelihoods strategies; these are for example, livestock husbandry, off-farm work, and cropping among others, resulting in outcomes such as more income, reduced vulnerability, and more sustainable natural resource based (15). Livestock is often considered a vital physical asset which can help people to be less vulnerable and even the means to step out of poverty (16).

**Figure 1:**
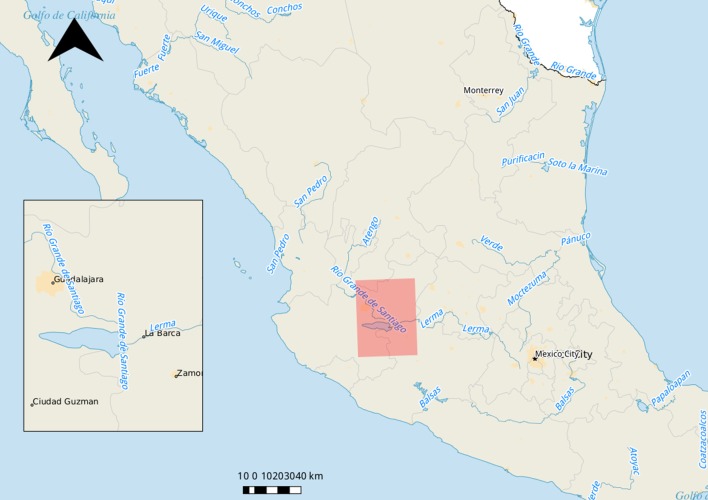
Localization of the research area. Source: own elaboration.

We have used mixed methodology of qualitative and quantitative methods to characterize goat farmers livelihoods and caprine brucellosis status in the Mexican Bajío within the states of Michoacán and Jalisco (17). We have chosen this region because is a goat dairy basin area within the Bajío of Mexico (Figure 1).

**Figure 2:**
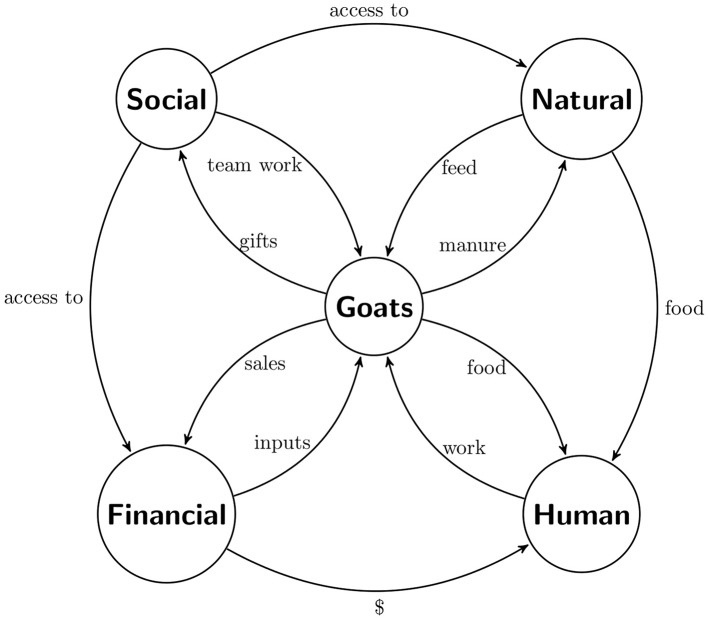
Flow chart of livelihood assets around goats. Source: own elaboration.

The Bajío is located in west central Mexico and has been characterized since colonial times by the quality of the crop land. Main crops are maize, wheat, beans, and chickpeas by the Goat husbandry played a key role in farmers livelihoods. Figure 2 shows a feedback system representation of goat husbandry. From a longitudinal survey with 46 farmers chosen by convenience we found that average number of dairy does in flock was 65. Does required human capital; this is farmers' care and work, in return farmers obtained animal protein from goats, i.e., milk and meat. The average milk yield was 0.80 liters per day per doe. Goat's milk was sold, which provided a weekly income.

Besides labor, a goat flock required inputs such as feed and veterinary drugs. Goats were integrated to the cropping system of the region and were herded to graze crop residues while their manure was a natural fertilizer. Having goats allowed people to work with others, farmers helped each other in their tasks by taking turns in grazing two or three flocks together. In addition goat kids were gifts to pay back for favors or services. Bajío farmers were often granted to herd their flocks to graze crop residues in neighbors' crop land. In return farmers made payment-in-kind with a goat kid (17). In general, goat farming popularity among resource-poor farmers of the region shows a revival contrasting with figures at national level where the number of goat farmers dropped by 40 between 1991 and 2007 (18, 19). Smallholder farmers of the Bajío are eager to keep goats for the following three reasons:

First, jobs scarcity; well-paid jobs in the region are scant and undocumented emigration to the USA has become riskier and tougher. A farmer said to us when we asked him why is that he became a goat farmer:

“Here in this village you either have goats or you go to the US; I was once an immigrant. I did not like it, this why I am a goat farmer.”

Farmers appreciate very much goat farming as it is a source of income. They said colloquially “it is better to herd than be herded” meaning that farmers are happier being their own boss rather than obeying someone else. Goat farming can be carried out by landless farmers.

Second, a growing demand for goat milk and cropping maize the main staple crop is less profitable. The demand for goat milk is triggered by the caramel industry. This industry monopolizes goat milk trade to make caramels sold in Mexico and in the US.

Third, the relative low cost of feeding goats. In the Bajío region of Michoacán and Jalisco goat husbandry is pre-dominantly a pastoral activity. Goats are herded to graze crops residues in the valleys during the dry season and native vegetation in the hills and mountains during the rainy season.

Besides goat farming other livelihood strategies are utilized by households to make a living, such as cropping, other livestock rearing (cattle, poultry, pigs, and sheep), off-farm work, and remittances (3).

## Brucellosis Control: Opportunities for Sustainable Livelihoods

A drawback of goat husbandry in the area is brucellosis endemicity in the goat population. Our serological survey in 1,768 goats showed a high brucellosis apparent prevalence in goats: 11–38% depending on the region (20). Not surprisingly, villagers avoid goat milk in their diet. Drinking goat milk causes fever farmers said. Moreover, brucellosis has afflicted farmers badly, according to the testimonies of two farmers one had a spinal involvement due to brucellosis and the other epididymo-orchitis.

Brucellosis infections in humans are shocks, which are part of the vulnerability context and have a negative effect on the human capital. Likewise brucellosis outbreaks in goat flocks diminishes farmers physical capital quality due to abortion, mastitis, infertility, and goat kid mortality (21). Hence, brucellosis control is an opportunity to reduce farmers' vulnerability, increase their income and well-being, improve food security, and provide a better natural resource base. We do not need to reinvent the wheel, enough technologies have been out for a long time. The key is then to understand farmers' limitations to adopt an effective brucellosis control strategy.

## Vaccination: Effects and Costs

Brucellosis in livestock can be controlled with the use of vaccines. The efficacy of vaccination in small-ruminants is well-documented (21–24). We found significant difference in the seroprevalence of goats depending on difference in vaccination: 38% in Jalisco (poorly vaccinated) and 11% in Michoacán (intensively vaccinated) (20).

It is generally accepted that vaccination is inexpensive, but this is not so for Mexican farmers. In Jalisco farmers willing to vaccinate their flocks were charged 10 Mexican pesos per goat [in 2008 the exchange rate of a Mexican peso (MX) was 0.08 US (25)]. While this price per goat vaccinated might be seen as inexpensive, it was not to the farmers who were used to vaccinate goats against other diseases, and these costs were about nine times cheaper compared to brucellosis vaccination. Furthermore, during the field work we noticed that freelance veterinarians in Jalisco rarely visited farmers to offer the vaccination and once a veterinarian vaccinated goats at the wrong time of the year; goats were pregnant when vaccinated, resulting in an abortion storm in some of the flocks [(15), p. 92].

So farmers can be reluctant to invest in vaccination being afraid for potential errors at vaccine application and because the unawareness of the benefits of brucellosis control can have through vaccination.

We have simulated the economic benefits of different control strategies for various scenarios (26). The simulation was run through a series of epidemiological and economic models. Our models have shown that a regional caprine brucellosis vaccination campaign can be economically rewarding for the goat sector (26). The cost/benefit ratio of vaccination was 3.8 MX and the net present value was 3.2 MX. We could not calculate the benefits on human health, to our knowledge this has not been evaluated yet in Mexico. Roth et al. (27) showed that the economic benefits of vaccination of cattle and sheep in Mongolia were substantial; three times higher than the cost of the intervention for the whole country in a 10 year-period (about 18 million USD), taking the impact on human health into account.

According to our models in Oseguera Montiel et al. (26) vaccination with Rev 1 does not eradicate brucellosis though it will reduce its prevalence. In brucellosis endemic goat populations, brucellosis vaccination on its own can lower the seroprevalence by two thirds in a 10 year-period. To lower the prevalence below 3%, which is the eradication phase according to the Mexican law, will need a test-and-cull strategy. Caprine brucellosis eradication, through test-and-cull is considered to be hard to achieve and is most unlikely to happen in developing countries. A possibility to apply a test-and-cull strategy could be to add value to goat milk by entering a different market through a value chain. Our research showed that test-and-cull approach becomes feasible in economic terms if farmers could sell their milk for a three times higher price (26).

Currently the goat milk processing industry does not pay more for milk coming from brucellosis free flocks, probably because goat milk is used for caramels and they see no risk of brucellosis for consumers as the pathogen does not survive the production process. Farmers' opportunity then is by negating the dairy industry, e.g., brucellosis control could offer an opportunity to create a new market. A potential marketing possibility could be to offer goat products directly to consumers by-passing middlemen, retailers, and the big dairy industry. Farmers in the study site are close to good potential markets, such as Guadalajara metropolis with 4 million people about 150 km from the villages. Farmers have to get involved in local initiatives promoting fair trade of their products which need to be of good quality and free of pathogens, especially *Brucella* spp.

## Participatory Brucellosis Control Strategy

It seems that little attention is given to the problems farmers in the global South encounter to implement brucellosis control strategies. Engagement of farmers in designing the control strategy is very much needed. Thus, a good understanding of farmers' practices, knowledge, interests, beliefs, and experiences is needed (28). We propose a strategy that takes into account the latter aspects.

First, help farmers to reach better markets. A positive feedback loop has to be sought, i.e., better quality and safe milk resulting in better cash returns to farmers. Farmers' main concern is the milk price, which does not keep up with the inflation. If they could get a better price for their milk when they have a brucellosis free flock, surely they will make the efforts to reach that status brucellosis away from their flocks.

Second, recognize farmers' knowledge and experience to gain their support. Various stakeholders such as milk buyers and state employees of the agricultural ministry (veterinarians, extensionists) showed low esteem of farmers and are arguing their lack of knowledge (29). Ethnographic methods (e.g., participatory observations and in-depth interviews) have shown, however, that farmers have a wealth of knowledge of the agro-ecology of the region, goat diseases, treatments, disease prevention, cropping, and economics (3). Farmers' knowledge explains partly why goat husbandry has persisted for almost 500 years and farmers have adapted production goals over time. And their knowledge will be useful for the design of brucellosis control strategies which are suited to farmers' socio-economic situation and agro-ecological context.

Third, Compensate farmers for their loss if a test-and-cull strategy. Financial support has been key for the success of brucellosis control campaigns in European countries (30). A proper test-and-cull strategy will bring down the prevalence more quickly. But test-and-cull will be very difficult to implement when farmers have to bear the costs on their own or when milk prices do not allow investment in test-and-cull.

Fourth, Make farmers more aware of all aspects related to brucellosis. While farmers have a wealth of knowledge on goat husbandry and some diseases including brucellosis there are some knowledge gaps to tackle. Especially farmers should be better informed about the implications of brucellosis in goats and ways to prevent infections in animals and humans. Broadcasting to inform farmers about brucellosis is an option, but there is a range of other possibilities. Smits (23) suggested the use of text messages in mobile phones, another possibility is to include some entertaining lectures about brucellosis for children in rural schools.

Fifth, Farmers could make use of their milk for their own use if they can boil their milk to minimize risks of brucellosis infection.

Sixth, A regional approach is needed to effectively control brucellosis. *Brucella* pathogens can spread beyond administrative borders (23). Brucellosis control in Mexico is organized within the boundaries of states. Direct and indirect transmission of brucellosis among goats of more than one state is possible and very likely as goats are traded between states or graze in border areas and in territories of two states alike.

## Conclusions

In developing countries such as Mexico, caprine brucellosis is a great constraint for smallholder goat farming development and a threat for public health and especially for farmers' livelihoods. Brucellosis control has proven to be complex, and goes beyond veterinary sciences. By an understanding of farmers assets, livelihoods strategies, and their environment a livelihoods perspective approach helps to understand how farmers can benefit when they implement brucellosis control strategies.

## Author Contributions

The manuscript summarizes DO's Ph.D. dissertation, which was supervised by AvdZ, HU, and KF.

### Conflict of Interest Statement

The authors declare that the research was conducted in the absence of any commercial or financial relationships that could be construed as a potential conflict of interest.
